# High-Throughput Chemotherapeutic Drug Screening System for Gastric Cancer (Cure-GA)

**DOI:** 10.1245/s10434-024-16850-0

**Published:** 2025-01-23

**Authors:** Jieun Lee, In Hee Kim, Donghyeok Seol, Sangjun Lee, Mira Yoo, Tae-Kyeong Lee, So Hee Yoon, Eunju Lee, Duyeong Hwang, So Hyun Kang, Young Suk Park, Bosung Ku, Sang Youl Jeon, Yongmun Choi, Keehoon Jung, Ji-Won Kim, Jin Won Kim, Sang-Hoon Ahn, Keun-Wook Lee, Hyung-Ho Kim, Hyeon Jeong Oh, Dong Woo Lee, Yun-Suhk Suh

**Affiliations:** 1 Department of Surgery, Seoul National University Bundang Hospital, Seongnam, Republic of Korea; 2 Department of Precision Medicine, Medical and Bio Decision (MBD Co., Ltd), Suwon, Republic of Korea; 3 Department of Surgery, Kyung Hee University Hospital at Gangdong, Seoul, Republic of Korea; 4 Department of Surgery, Chung-Ang University Gwangmyeong Hospital, Gwangmyeong, Republic of Korea; 5 Department of Anatomy and Cell Biology / Biomedical Sciences, Seoul National University College of Medicine, Seoul, Republic of Korea; 6 Department of Internal Medicine, Seoul National University Bundang Hospital, Seongnam, Republic of Korea; 7 Department of Pathology, Seoul National University Bundang Hospital, Seongnam, Republic of Korea; 8 Department of Biomedical Engineering, Gachon University, Seongnam, Republic of Korea; 9 Department of Surgery, Seoul National University College of Medicine, Seoul, Republic of Korea

**Keywords:** Tumoroids, High-throughput screening (HTS), Patient-derived cancer cell, Multiparameter index (MPI), Drug response prediction, Gastric cancer

## Abstract

**Background:**

Three dimensional (3D) cell cultures can be effectively used for drug discovery and development but there are still challenges in their general application to high-throughput screening. In this study, we developed a novel high-throughput chemotherapeutic 3D drug screening system for gastric cancer, named 'Cure-GA', to discover clinically applicable anticancer drugs and predict therapeutic responses.

**Methods:**

Primary cancer cells were isolated from 143 fresh surgical specimens by enzymatic treatment. Cell-Matrigel mixtures were automatically printed onto the micropillar surface then stabilized in an optimal culture medium for 3 days to form tumoroids. These tumoroids were exposed in the drug-containing media for 7 days. Cell viability was measured by fluorescence imaging and adenosine triphosphate assays. On average, 0.31 ± 0.23 g of fresh tumor tissue yielded 4.05×10^6^ ± 4.38×10^6^ viable cells per sample.

**Results:**

Drug response results were successfully acquired from 103 gastric cancer tissues (success rate = 72%) within 13 ± 2 days, averaging 6.4 ± 2.7 results per sample. Pearson correlation analysis showed viable cell numbers significantly impacted drug data acquisition (*p *< 0.00001). Tumoroids retained immunohistochemical characteristics, mutation signatures, and gene expression consistent with primary tumors. Drug reactivity data enabled prediction of synergistic drug correlations. Additionally, a multiparameter index-based prognosis model for patients undergoing gastrectomy followed by adjuvant XELOX was developed, showing significant differences in 1-year recurrence-free survival rates between drug responders and non-responders (*p* < 0.0001).

**Conclusions:**

The Cure-GA platform enables rapid evaluation of chemotherapeutic responses using patient-derived tumoroids, providing clinicians with crucial insights for personalized treatment strategies and improving therapeutic outcomes.

**Supplementary Information:**

The online version contains supplementary material available at 10.1245/s10434-024-16850-0.

According to the standard guidelines of the Korean Society for Gastric Cancer, the first-line treatment for stage II and III patients is surgery followed by adjuvant chemotherapy, and systemic chemotherapy is recommended for stage IV patients.^1^ Adjuvant XELOX (capecitabine plus oxaliplatin) significantly improves the disease-free survival of patients with stage II–III resectable gastric cancer (GC) compared with D2 gastrectomy alone.^2^ However, 56.3% (182/323) of patients are expected to be resistant to the XELOX regimen, suggesting that adjuvant chemotherapy is not effective for all patients with resectable GC.^3^ Therefore, it is important to predict response to chemotherapy to suggest an appropriate anticancer drug after surgery for patients with advanced GC (AGC). Previous studies have suggested biomarker genes for predicting response to chemotherapy in patients with GC^3^ but the results were based on retrospective cohort-based association analysis rather than on biological or molecular experiments for each patient. Therefore, a more flexible drug-responsive evaluation platform covering multiple drugs is needed for AGC.

Since the discovery that trastuzumab improves the prognosis of patients with human epidermal growth factor receptor 2 (HER2) overexpressing GC,^4^ many targeted therapies have been developed but the results have been insufficient.^5–7^ AGC shows varying responses to anticancer drugs with different biological properties within a single tumor due to increasing intratumoral heterogeneity,^8,9^ limiting the effectiveness of uniform treatments. This tumor heterogeneity significantly affects response to traditional, non-targeted anticancer drugs. Individual patients can have varying responses to the currently used, empirically chosen anticancer drugs, leading to disparities in prognosis.

High-throughput screening (HTS) systems allow for rapid and cost-effective simultaneous testing of numerous potential drugs against various cancer cells.^10,11^ While this approach has been successful, the precise prediction and interpretation of therapeutic effects of drugs are limited in terms of sensitivity and selectivity because the two-dimensional (2D) cell growth environment does not mimic native tissue.^12^ Animal models can emulate physiological complexity at the whole-organism level but they lack scientific validity and translatability to human disease models.^12,13^ Three-dimensional (3D) cell cultures are useful for personalized/precision medicine and corresponding drug screening as they can mimic physiological and pathological conditions while eliminating the discrepancies between animal and human models.^14^ Additionally, as an in vitro platform, 3D cell cultures can be integrated with imaging,^15^ automation,^16^ and computational tools,^17^ making them a promising next-generation method for effective and precise drug discovery and development. In addition, cell lines stabilized through subculture often lose the biological characteristics of their original tissue due to changes in morphology, genetic mutations, and gene expression during culture.^18^ In this respect, several research groups are working on ‘tumoroids’ (tumor-like organoids), which are typically derived from primary tumors harvested from patients.^19,20^

In this study, we developed a high-throughput chemotherapeutic drug screening system for GC, named the ‘Cure-GA’ platform, and used the screening results to establish a multiparameter index (MPI)-based prediction model. The main aim of our platform was to evaluate the response of patient-derived primary cancer cells to various anticancer drugs within 2 weeks after surgery and to utilize ex vivo drug response databases to predict patient responses to chemotherapy.

## Materials and Methods

### Human Gastric Cancer Tissues

Stomach tumor specimens were collected from patients at Seoul National University Bundang Hospital (2021–2022), with informed consent and Institutional Review Board (IRB) approval (B-2003-603-303). Histopathological assessment confirmed tumor presence and purity by an independent histopathologist. Fresh tumor tissue, approximately 5×5×5 mm from multiple sites, was collected on the day of surgery. About 15% was preserved as a paraffin-embedded block for tumor purity evaluation. If surgery occurred in the morning, tissue collection and cell isolation were performed on the same day. For surgeries later in the day, harvested tissue was stored in a tissue storage solution (Miltenyi Biotec #130-100-008) and refrigerated overnight.

### Cure-GA System

For tissue dissociation, fresh surgical tissues were collected from multiple sites within the recommended area for tumor tissue sampling from one resection specimen, minced in sterile Petri dishes, and processed in a GentleMACS C tube (Miltenyi Biotec #130-093-237) with a tumor dissociation kit (Miltenyi Biotec #130-095-929) according to the manufacturer’s protocol. The tubes were subjected to the 37_h_TDK_1 protocol using a GentleMACS Dissociator. Dissociated cells were filtered through a 70 µm MACS Smart Strainer (Miltenyi Biotec #130-110-916) and washed with Roswell Park Memorial Institute (RPMI) 1640 medium; red blood cells (RBCs) were lysed with a specific RBC lysis buffer (BioLegend #420301). Cell viability and total cell counts were assessed using Trypan blue (Invitrogen #T10282) and a Countess II Automated Cell Counter (ThermoFisher).

For drug screening, dissociated cells were automatically dispensed on a 384-Pillar plate (Cellvitro^®^ 384PM, MBD, Suwon, Korea) using ASFA^®^ Spotter V6 (MBD-SP-HE006, MBD) with a density of 10,000 cells in 1.5 μL mixed with 70% Matrigel per spot. In our experiments, Matrigel was utilized for 3D cell culture. Previous studies^21–23^ have emphasized the impact of Matrigel on cell proliferation and the permeability of drugs and nutrients in 3D culture systems. These effects are pivotal in 3D cancer cell culture models, facilitating the establishment of more clinically relevant, patient-like models. The gelation process was conducted in a 37 °C incubator for 1 h. Following gelation, the chips were combined with a modified 384-well plate containing a specific tumoroid culture medium enriched with various growth factors such as epidermal growth factor (EGF), noggin, R-spondin1, fibroblast growth factor 10 (FGF10), gastrin, and a transforming growth factor (TGF)-β inhibitor (electronic supplementary material [ESM] Table S1). To prevent drying, the 384 chip sets were placed in an MBD chamber and incubated for 72 h. Cells were then treated with drugs in a fresh GC tumor medium and incubated for an additional 7 days at 37 °C in a 5% CO_2_ incubator.

### Cell Viability Assessment and Data Analysis

Tumoroids were stained using calcein AM, and images were captured at various z-focus positions with an ASFA^®^ Scanner V5 ST (MBD-SC-ST005, MBD). The images underwent deconvolution for 3D visualization, and growth rates were analyzed using mean areas of the cultured cells. Cell viability was assessed through adenosine triphosphate (ATP) monitoring (CellTiter-Glo^®^ Cell Viability Assay; Promega, Madison, WI, USA), and luminescence was measured with a Spark microplate reader. Dose-response curves were generated to determine the half maximal inhibitory concentration (IC_50_) and area under the curve (AUC) values using non-linear regression analysis in GraphPad Prism 9 (GraphPad Software, La Jolla, CA, USA), with statistical significance evaluated using an unpaired Student’s *t* test.

**Fig. 1:**
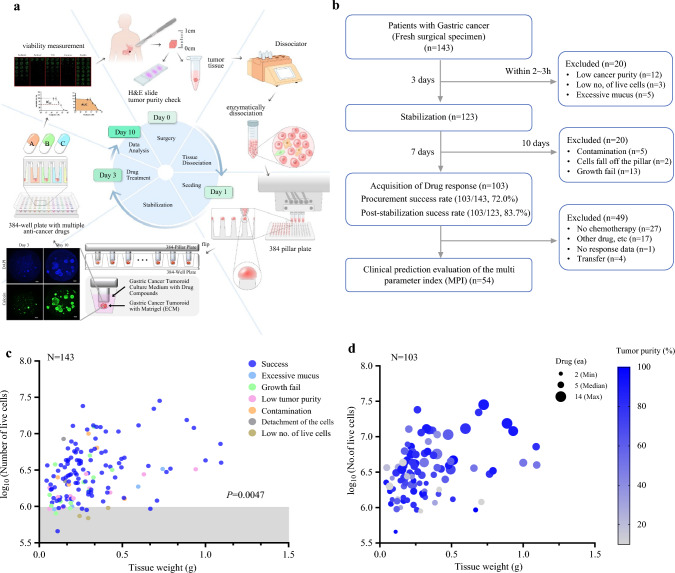
Workflow and performance of the Cure-GA platform. **a** Schematic workflow of the Cure-GA platform, from sample preparation to drug response evaluation, completed within 10 days using patient-derived primary cancer cells. **b** Flow diagram of drug response data acquisition. Of 143 fresh GC tissue samples, 12 were excluded due to low tumor cell purity. Additional exclusions were made for contamination, cell detachment, or growth failure, yielding datasets for 103 patients (72% success rate). **c** Approximately 4.1 × 10^6^ cells were isolated from an average of 0.3 g of tissue. The success rate was 75% when viable cell counts exceeded 1 × 10^6^ (*p* = 0.0047, Chi-square test). Gray shading indicates samples with fewer than 1 × 10^6^ cells. **d** Relationship between tumor purity and the number of successful drug datasets. Circle size reflects the number of drugs tested per sample, and color gradient indicates tumor purity. *H&E* hematoxylin and eosin, *ECM* extracellular matrix, *GC* gastric cancer

The AUC index is the AUC value converted to a standard score (*Z* score) and was calculated as follows using the mean and standard deviation (SD) of the AUC. All procedures, including drug response data reports, were completed within 13 ± 2 days after surgery (Fig. 1, ESM Fig. S1) [Eq. 1].

1 $${\text{AUC Index}}_{\text{drug}}=Z{\text{ score of AUC}}_{\text{drug}} = \frac{{\text{AUC }}_{\text{drug}}-\text{mean}}{\text{SD}}$$

### Multiparameter Index-Based Prediction Model

To develop a predictive model, 1-year recurrence data from 54 patients treated with XELOX were analyzed. Patients were classified as clinical responders or non-responders based on their recurrence status. We then selected the AUC data derived from either oxaliplatin- or 5-fluorouracil-treated tumoroids from the established database; we selected the drug that induced the greatest response in tumoroids corresponding to each patient.^24,25^ AUC data from drug-treated tumoroids were selected to predict responses. Multiple logistic regression analysis was performed using *Z* scores of AUC, TNM stage, and growth rate as independent variables, with the recurrence information as the dependent variable (Eq. 2).

2 $${\text{Multi-parameter index}}_{\text{drug}}={Z\text{ score of }}Y_{\text{drug}}= \frac{{Y}_{\text{drug }}-\text{ mean}}{\text{SD}}$$

The MPI was defined to differentiate between drug responders and non-responders, establishing a cut-off value of 0.3 for optimal discrimination.

The predictive model's effectiveness was assessed through sensitivity, specificity, accuracy, positive predictive value (PPV), and negative predictive value (NPV). Formulas for these metrics were outlined, with true positive, false positive, false negative, and true negative defined in the context of the study.

### Hematoxylin and Eosin Staining and Immunohistochemistry

Tumor tissues were fixed in 10% formalin, paraffin-embedded, and sectioned at 4 μm. Tumoroids were fixed in 4% paraformaldehyde (30 min, RT) and embedded in Histogel (Epredia, Kalamazoo, MI, USA). Hematoxylin and eosin (H&E) staining and immunohistochemistry (IHC) were conducted using a BenchMark ULTRA IHC/ISH System (Roche) with an OptiView DAB Detection Kit. Briefly, sections were deparaffinized (EZ Prep, 76 °C, 4 min), underwent antigen retrieval (CC1, 100 °C, 24 min), and peroxidase inhibition (37 °C, 4 min). Slides were incubated with primary antibodies against anti-EpCAM (Santa Cruz #SC-25308, 1:100), anti-p53 (DAKO clone Do-7, 1:1000), and anti-HER2 (VENTANA clone 4B5, 1:1000) at 37 °C for 16 min, followed by detection with HQ Universe linker/HRP Multimer and DAB. Counterstaining with hematoxylin and bluing reagent (37 °C) was performed and images were scanned with Motic EasyScan.

### Whole-Exome Sequencing, RNA Sequencing, and Data Analysis

Frozen tumor tissue, matched normal tissue, and tumoroid tissue were ground in liquid nitrogen, and genomic DNA was extracted using a QIAamp DNA Mini Kit (Qiagen, Cat# 51306). Whole-exome capture was performed on all samples with the SureSelect Human All Exon V6 Kit (Agilent Technologies, Tokyo, Japan). The captured targets were subjected to 101 bp sequencing using a HiSeq 2500 (Illumina, San Diego, CA, USA). Total RNA was isolated from normal/tumor tissues and tumoroid lysates using TRIzol (Qiagen) and a Qiagen RNeasy kit (Qiagen, Cat# 74134). A TruSeq Stranded mRNA Library Prep Kit (Illumina, Cat #RS-122-2101) was used for library construction. Paired-end sequencing reads of cDNA libraries (101 bp) were generated using a NovaSeq 6000 (Illumina, San Diego, CA, USA). The produced reads were trimmed for adapter sequences and low-quality sequences using Trim Galore (v0.6.9). Detailed data analysis methods are described in the Methods section of the ESM.

## Results

### Workflow and Performance of the Cure-GA Platform

We designed the Cure-GA system using patient-derived primary GC cells obtained from surgically resected fresh GC tissue (Fig. 1a). About 5×5×5 mm of fresh tumor tissue was obtained in the operating room on the day of the gastrectomy. The average tissue weight obtained from the 143 enrolled patients was 0.31 ± 0.23 g. Primary cancer cells were isolated from GC tissues within the same or following day and applied to the Cure-GA platform (Fig. 1b). We obtained drug response data from 103 patient tissues with a 72% success rate (103/143). Among the 40 unsuccessful cases, 12 were excluded due to cancer cell purity being <10% based on pathological evaluation. Cases with insufficient cell counts (< 6.4×10^5^ cells) or elevated viscosity, which prevented printing, were also excluded. Additionally, instances of contamination during culture, cell detachment from the pillar surface due to overgrowth, or growth failure were omitted from the data analysis. Initial experiments faced contamination issues but these were resolved in subsequent trials by adding 0.2% amphotericin B to the culture medium.

The average weight of the tissues used in this Cure-GA was 0.31 ± 0.23 g (0.04–1.1 g), and the average total live cell count after tissue dissociation was 4.05×10^6^ ± 4.38×10^6^ cells (4.5×10^5^–2.8×10^7^ cells). When the viable cell count was 1×10^6^ or more, the success rate reached 75.4%. We used 1×10^6^ cells/tissue sample as the standard and achieved stable drug response data with a live cell rate of at least 1×10^6^ cells per tissue (*p *= 0.0047) [Fig. 1c]. Although the success rate increased with tissue weight, there was no significant correlation between the tissue weight and the success rate (*p *= 0.2727).

The number of cells needed to ensure assay robustness was 10,000 cells per pillar in a 384-well plate. As the cell-Matrigel mixtures were printed onto the predefined location of the micropillar surface, at least 2.1×10^5^ live cells (three repetitions of seven concentrations per drug) were used per drug. For the 103 tissues for which drug response data were successfully obtained, the average tumor purity was 70% ± 23.4%.

An average of 6.4 ± 2.7 (2–14 drugs/tissue) drug responsiveness datasets were collected for each tissue (Fig. 1d). Eleven percent of patients had sufficient live cell numbers to obtain response data for 10 or more drugs but 12% had results for fewer than four drugs due to cell number limitations. In most cases, drug response results were obtained for more than 4 to < 10 (77%) chemotherapeutic drugs per patient (ESM Fig. S2a). This means that the greater the number of cells available, the more types of drugs can be tested. Additionally, Pearson correlation analysis across tissue weight, number of live cells, tumor purity, and obtained drug data showed that the correlation between acquired drug data and number of live cells was the highest (*p *< 0.00001), and tissue weight and number of live cells also showed a significant correlation (*p *= 0.00004) [ESM Fig. S2b]. On the other hand, tumor purity had a relatively low correlation with other variables. Tumor purity was determined by pathological evaluation and was used as a reference in the data analysis. Table 1 presents the clinicopathologic characteristics of a cohort comprising 103 successful cases and 40 unsuccessful cases. No differences in clinicopathologic background were observed between the two groups.

**Table 1:** Clinicopathologic characteristics of the cohorts [*N* = 143]

Category	The 103 successful cases [*n *= 103]	The 40 unsuccessful cases [*n *= 40]	*p* value^a^
*Age*, years [median (range)]	64 (30–87)	62.5 (41–78)	0.9206
*Sex*			0.09822
Male	69 (67)	33 (83)	
Female	34 (33)	7 (18)	
*Lauren's classification*			0.7358
Intestinal	40 (39)	17 (43)	
Diffuse	48 (47)	17(43)	
Mixed	1 (1)	0	
Indeterminate	14 (14)	3 (8)	
NA	0	3 (8)	
*Ming's classfication*			1
Infiltrative	92 (89)	34 (85)	
Expanding	10 (10)	3 (8)	
NA	1 (1)	3 (8)	
*T stage*			0.2107
T1	5 (5)	6 (15)	
T2	16 (16)	4 (10)	
T3	40 (40)	15 (38)	
T4	41 (41)	13 (33)	
NA	1 (1)	2 (5)	
*N stage*			0.1553
N0	20 (19)	16 (40)	
N1	15 (15)	5 (13)	
N2	23 (22)	5 (13)	
N3a	21 (20)	8 (20)	
N3b	23 (22)	6 (15)	
NA	1 (1)	0	
*M stage*			0.71
M0	97 (94)	37 (93)	
M1	6 (6)	3 (8)	
*TNM stage*			0.3678
I	10 (10)	7 (18)	
II	27 (26)	11 (28)	
III	60 (58)	17 (43)	
IV	6 (6)	3 (8)	
NA	0	2 (5)	
*Differentiation*			0.551
Papillary	5 (5)	0	
Well differentiated	2 (2)	1 (3)	
Moderately differentiated	31 (30)	16 (40)	
Poorly differentiated	25 (24)	8 (20)	
Mucinous	2 (2)	3 (8)	
Poorly cohesive carcinoma	18 (17)	5 (13)	
Mixed	12 (12)	4 (10)	
Other^b^	8 (8)	3 (8)	
*MSI*			0.4385
MSI-H	11 (11)	5 (13)	
MSI-L	7 (7)	4 (10)	
MSS	85 (83)	25 (63)	
NA	0	6 (15)	
*EBV*			1
Negative	98 (95)	39 (98)	
Positive	5 (5)	1 (3)	
*HER2 IHC*			0.7246
Negative	76 (74)	30 (75)	
Positive	8 (8)	2 (5)	
NA^c^	19 (18)	8 (20)	
*Tissue ischemic time*^d^			0.423
On the day of surgery	36 (25)	16 (40)	
Within 24 h	65 (63)	22 (55)	
Within 48 h	2 (2)	2 (5)	

Data are expressed as *n* (%) unless otherwise specified

*NA* not available, *MSI* microsatellite instability, *MSI-H* MSI-high, *MSI-L* MSI-low, *MSS* microsatellite stable, *EBV* Epstein–Barr virus, *HER2* human epidermal growth factor receptor 2, *IHC* immunohistochemistry

^a^The Fisher’s exact test and Mann–Whitney U test were used for the statistical significance test of categorical and continuous variables, respectively

^b^Gastric carcinoma with lymphoid stroma (3), gastric adenocarcinoma with enteroblastic differentiation (1), hepatoid adenocarcinoma (1), small cell neuroendocrine carcinoma (2), Omentum metastasis (1)

^c^HER2 IHC 2+ but need more validation

^d^The time from harvesting the tissue and placing it in a cold tissue storage solution until cell isolation

### Gastric Cancer Tumoroid Cells Maintain Patient Tumor Characteristics

**Fig. 2:**
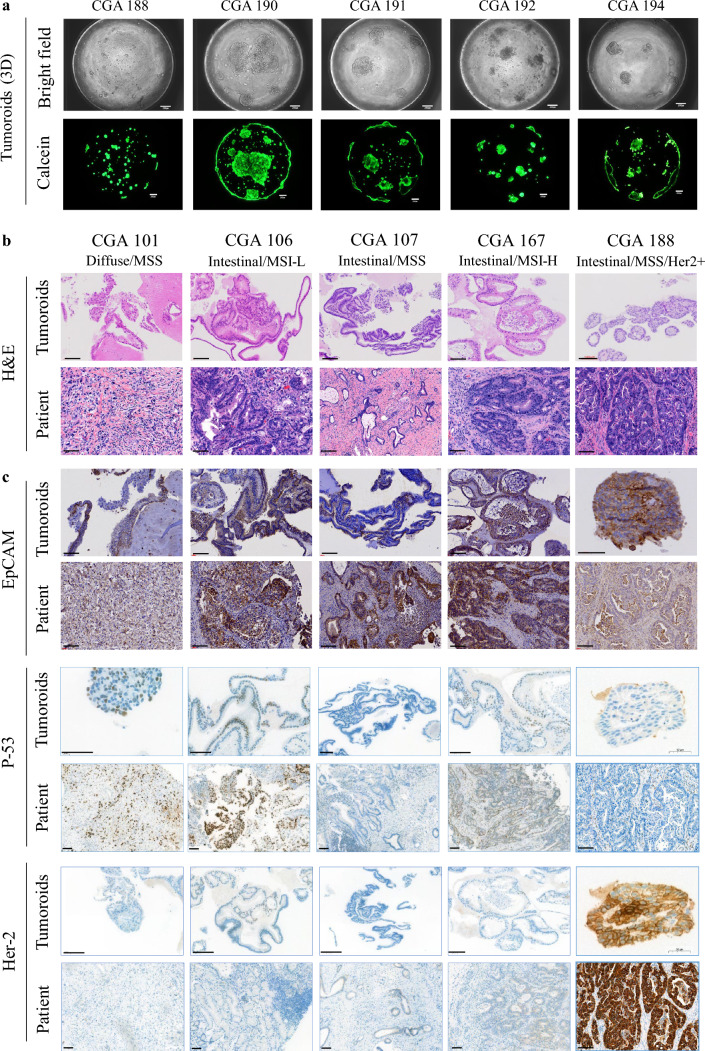
Tumoroids maintain primary tumor characteristics. **a** Bright field microscopy images and calcein AM-stained images of the cells on the micropillar surface on the 7th day after incubation in the well plate. Representative images of **b** H&E staining and **c** immunohistochemistry for the indicated antibodies of tumoroids and their primary tumor tissues. The scale bar is 100 μm. *H&E* hematoxylin and eosin, *3D* three-dimensional, *MSI* microsatellite instability, *MSI-H* MSI-high, *MSI-L* MSI-low, *MSS* microsatellite stable, *HER2* human epidermal growth factor receptor 2

Figure 2a shows microscopy images and calcein-stained images of tumoroids grown on micropillars for 10 days, demonstrating self-organization into multicellular structures such as balls and plates. H&E staining and IHC assays confirmed that tumoroids retained primary tumor properties. Tumoroids derived from diffuse-type (CGA 101) and intestinal-type (CGA 106, 107, 167, 188) cancers exhibited growth patterns similar to paired primary tissues (Fig. 2b). Immunohistochemical profiles were also consistently retained in tumoroids compared with those in primary tumors. Epithelial cell adhesion molecule (EpCAM), a known epithelial cell marker,^26^ was expressed in all CGA cancer tissues and their tumoroids. Cellular tumor antigen p53 (P53) was highly expressed in primary cancer tissues of CGA101 and 106, and these expression patterns were reproduced in their tumoroids. CGA188 shows strong HER2 expression, similar to that in primary tumors (Fig. 2c). These results indicate that patient-derived tumoroids, predominantly composed of epithelial cells, effectively recapitulate the immunohistochemical characteristics of the primary tumors.

### Mutation and Expression Landscape of Gastric Cancer Patient-Derived Tumoroids

**Fig. 3:**
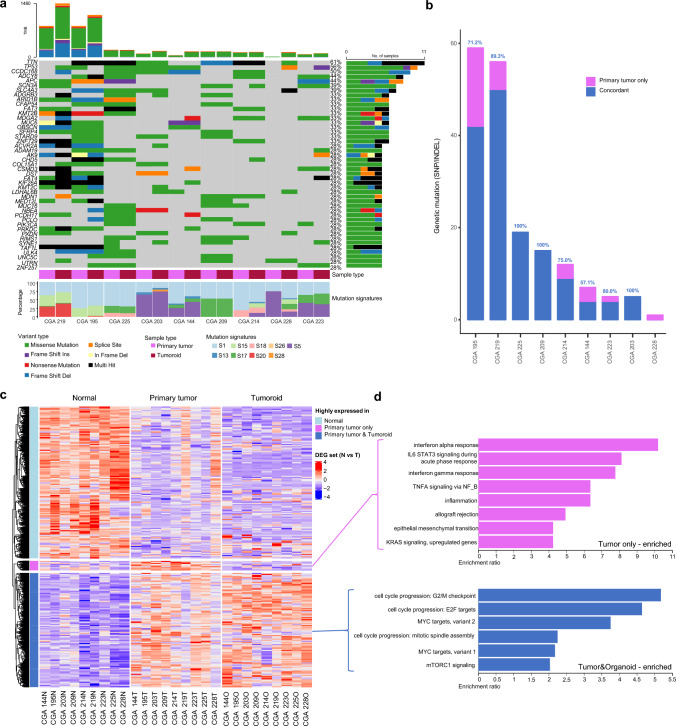
Mutation and expression landscape of GC patient-derived tumoroids. **a** Mutations and mutation signatures of each primary tumor tissue and paired tumoroids. Mutations detected in more than five samples are presented. **b** The number of concordant mutations in tumoroids among mutations detected in primary tumors. Only mutations of 205 genes included in the COSMIC Cancer Gene Consensus were used. **c** Heatmap showing normalized expression patterns of DEGs between normal and primary tumor samples (*p*_adj_ < 0.01). **d** Overrepresentation analysis using the DEG list from **c** [*p*_adj_ < 0.05]. *GC* gastric cancer, *DEGs* differentially expressed genes

To address whether patient-derived tumoroids reflect the molecular characteristics of parental tumors and inform chemotherapeutic strategies, mutation and gene expression analyses were performed. Initial quality control showed that each patient dataset comprising six pairs of sequencing data (DNA/RNA-normal, tumor, and tumoroid) was correctly labeled (ESM Fig. S3). The sample-specific mutation and mutation signatures of each primary tumor tissue sample were well maintained in the tumoroids (Fig. 3a; ESM Figs. S4a, b). Due to limited DNA/RNA yield from micropillar-grown tumoroids, nucleic acids were extracted for sequencing after 1–5 passages. Whole-exome sequencing at average depths of 87X (normal), 281X (tumor), and 226X (tumoroid) revealed that mutation signatures of primary tumors overlapped with those of the tumoroids by more than 73%, except for CGA 228 (Fig. 3a). Among COSMIC Cancer Gene Consensus genes, seven tumoroids retained over 70% of parental tumor mutations, while two (CGA144 and CGA228) retained < 60% (Fig. 3b). Copy number alteration (CNA) patterns were also preserved, with Pearson correlations of 63–99%, except for CGA228 (ESM Fig. S4c), which showed hyper-CNA in tumoroids (ESM Fig. S5). Lower concordance rates were associated with reduced tumor purity and increased passage numbers (ESM Fig. S6).

Transcriptomic analysis demonstrated that tumoroids retained tumor-specific gene expression profiles. Tumoroids reflected the differential expression of 995 overexpressed and 1322 downregulated genes in tumor tissues relative to normal tissues (Fig. 3c). Overrepresentation analysis identified enrichment in pathways related to the G2/M checkpoint (False Discovery Rate (FDR) < 2.2e−16), Myc targets (FDR = 0.00032), and mTORC1 signaling (FDR = 0.00168) [Fig. 3d]. Additionally, patient-specific highly upregulated genes were highly expressed in the corresponding tumoroids in each patient (ESM Fig. S7). However, 82 genes, enriched in interferon-α response (FDR = 0.00008) and epithelial–mesenchymal transition pathways (FDR = 0.01484), were expressed exclusively in tumors but not in tumoroids.

### Evaluation of Drug Response to Various Anticancer Drugs Using Cure-GA

**Fig. 4:**
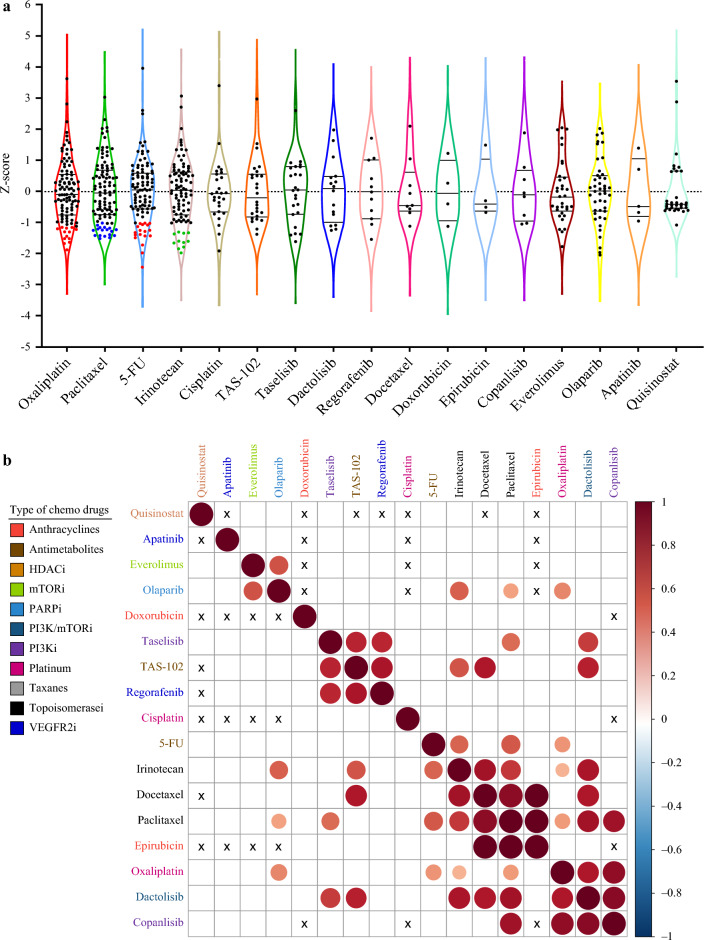
Tumoroids show divergent therapeutic responses to conventional chemotherapeutics. Response to 17 anticancer therapeutics was evaluated in the tumoroids of 103 GC patients. **a** Violin plot of normalized AUCs for 17 drugs in tumoroids from 103 GC patients. Horizontal lines indicate 0.25, 0.50, and 0.75 quantiles. Red, blue, and green dots represent Z scores for oxaliplatin/5-fluorouracil (IC_50_ < 2 μM), paclitaxel (IC_50_ < 100 nM), and irinotecan (IC_50_ < 200 nM), respectively. **b** Synergistic correlations between drugs based on AUC Z scores. Significant correlations (*p* < 0.05) are plotted, with pairs involving fewer than three cases marked as ‘X’. Dark brown indicates the strongest synergy. *GC* gastric cancer, *AUCs* area under the curves, *IC*_*50*_ half maximal inhibitory concentration, *AUC* area under the curve, *5-FU* 5-fluorouracil

Figure 4a presents drug response data as AUC *Z*-scores for tumoroids derived from 103 patients. Responses to commonly used GC chemotherapy agents, i.e. oxaliplatin, paclitaxel, 5-fluorouracil, and irinotecan, were evaluated alongside targeted drugs, including olaparib (poly[ADP ribose] polymerase [PARP] inhibitor), quisinostat (histone deacetylase [HDAC] inhibitor), and everolimus (mammalian target of rapamycin [mTOR] inhibitor). For oxaliplatin, the drug-sensitive group (IC_50_ ≤ 2 μM) clustered at the lower end of the violin plot (red dots), with similar clustering observed for paclitaxel, 5-fluorouracil, and irinotecan. Conversely, drug-resistant cases, including some relapsed patients, were concentrated at the upper end of the plots.

Clustering analysis was conducted to assess correlations between clinical factors (e.g., sex, age, TNM stage) and responsiveness to 5-fluorouracil, irinotecan, paclitaxel, and oxaliplatin (ESM Fig. S8). No clinical factors reliably distinguished drug resistance from sensitivity, highlighting the need for individualized drug reactivity assessments. Notably, a comparison of clinicopathologic features between 16 patients with recurrence within 1 year and 80 patients without recurrence revealed a significant association with WHO and Lauren classifications. Recurrence was more frequent in patients with poorly cohesive carcinoma and diffuse-type cancer (ESM Table S2).

Figure 4b shows the correlation matrix of drug responses across various chemotherapeutic agents categorized by their mechanisms of action, as highlighted by the color-coded legend. Each cell represents the correlation coefficient between two drugs, with the intensity and hue of the color indicating the strength and direction of the correlation (red indicates a strong positive correlation; blue indicates a strong negative correlation). Strong positive correlations to 5-fluorouracil, a baseline chemotherapeutic drug in GC, were observed in irinotecan, paclitaxel, or oxaliplatin. These correlations support currently established combination regimens such as SOX, XELOX, FOLFOX or FOLFIRI, or other second-line chemotherapies based on paclitaxel. Additionally, the PARP inhibitor olaparib also showed strong correlations with irinotecan, paclitaxel, and oxaliplatin, suggesting potential synergistic effect for novel combination regimens. However, careful consideration of potential drug-related adverse effects is necessary for strongly synergistic correlations, such as those observed between docetaxel, paclitaxel, and epirubicin, or between oxaliplatin and dactolisib or copanlisib. These findings suggest that incorporating synergistic drug combinations informed by Cure-GA data could enhance the effectiveness of treatment regimens, aligning with current guidelines for GC therapy.

### Comparison of the Area Under the Curve and Multiparameter Indices for Prediction of Anticancer Drug Response in Patients

**Fig. 5:**
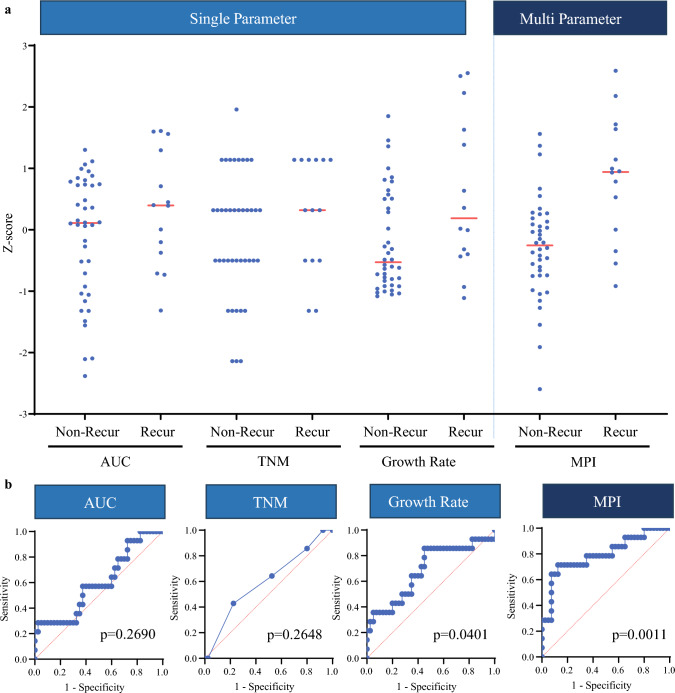
Comparison of Z scores across individual parameters for XELOX sensitivity analysis. **a** The Z scores of AUC, TNM, growth rate, and the MPI were plotted for patient response to XELOX (non-recurrence vs. recurrence). **b** The area under the ROC curves corresponding to the different parameters are shown. *AUC* area under the curve, *TNM* tumor, node, metastasis, *MPI* multiparameter index, *ROC* receiver operating characteristic

We evaluated the predictive capability of the established screening database for chemotherapy responses. AUCs for oxaliplatin- and 5-fluorouracil-treated tumoroids were compared with the clinical outcomes of 54 patients receiving postoperative XELOX. The red points on the graph in ESM Fig. S9 represent patients who received XELOX after surgery as a postoperative regimen and who were eventually diagnosed with clinical disease with recurrence. While single parameters such as AUC, TNM stage, or growth rate did not significantly correlate with recurrence, an MPI revealed a significant difference in *Z*-scores between the recurrence and non-recurrence groups (*p* = 0.0001) [Fig. 5a].

A logistic regression algorithm incorporating AUC, TNM stage, and growth rate (*Y* = 1.012*X*1 + 0.6454*X*2 + 1.211*X*3 – 1.404) achieved a receiver operating characteristic AUC of 0.7964 (*p* = 0.0011) [Fig. 5b]. Using an MPI cut-off of 0.3, clinical outcomes of 44/54 patients matched MPI predictions, with sensitivity, specificity, accuracy, PPV, and NPV of 85%, 71.4%, 81.5%, 89.5%, and 62.5%, respectively (ESM Table S3).

**Fig. 6:**
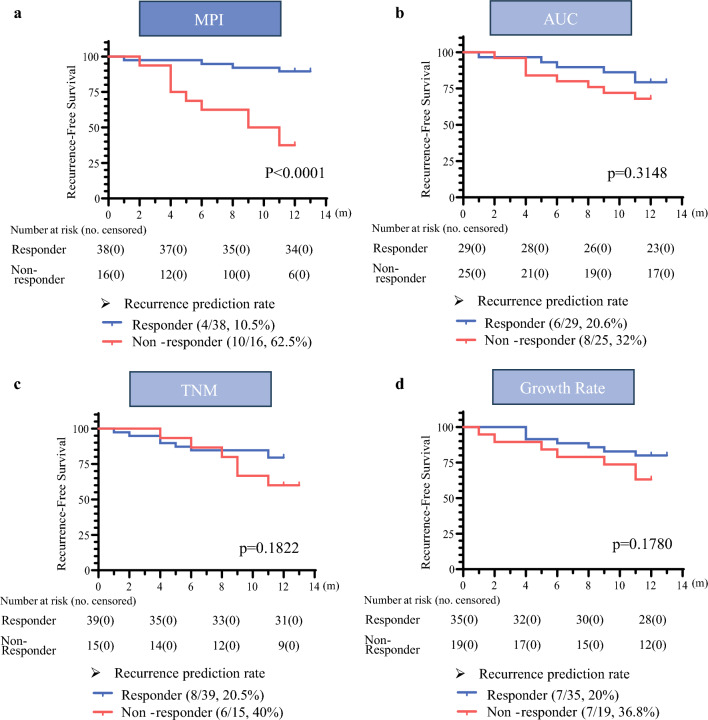
Comparison of 1-year recurrence-free survival across individual parameters for XELOX sensitivity. Kaplan–Meier survival curves were used to evaluate differences between drug responder and non-responder groups classified by individual parameters. **a** AUC; **b** TNM; **c** growth rate; **d** MPI. *AUC* area under the curve, *TNM* tumor, node, metastasis, *MPI* multiparameter index

Kaplan–Meier analysis of 1-year recurrence-free survival (RFS) demonstrated a significant difference between MPI-classified drug responder and non-responder groups (hazard ratio 0.06965, 95% confidence interval 0.02007–0.2417, *p* < 0.0001) [Fig. 6a]. When each single index was used to classify the drug responder and non-responder groups, a statistically significant difference between the groups was not observed (Fig. 6b–d). These results indicate that the MPI-based model effectively predicts chemotherapy responses and 1-year RFS in GC patients.

## Discussion

The rapid advancement of 3D culture technologies has greatly enhanced the compatibility of cultured cells with primary tissues, facilitating their application in cancer modeling and personalized medicine. In this study, we introduced the Cure-GA platform, a 3D culture-based drug screening system designed to evaluate anticancer drug responses in GC. Using patient-derived primary cancer cells, Cure-GA generated drug evaluation data within 10 days, achieving a success rate of 72% overall and 83.7% post-stabilization for 143 primary cell samples. By closely mimicking the molecular and phenotypic characteristics of primary tumors, the platform ensures high clinical relevance and provides a robust tool for HTS to identify patient-specific therapeutic options.

Unlike traditional 2D culture systems, which rely on cancer cell lines and reflect only limited tumor characteristics, tumoroid-based drug screening offers superior accuracy in predicting drug responses. Leveraging an organospotter-integrated high-TOP system, Cure-GA uses a standardized platform for high-throughput drug screening on micropillar plates. This enables rapid and comprehensive evaluation of individual responses to a wide range of chemotherapy and targeted agents, even identifying options in cases where standard clinical predictors fail. Additionally, the incorporation of MPIs enhances predictive accuracy for clinical outcomes, including RFS, compared with single-parameter assessments. The platform’s ability to incorporate molecular profiling also supports the identification of synergistic drug combinations, optimizing therapeutic efficacy.

However, the Cure-GA platform has limitations. First, the high cost of drug sensitivity testing may pose a challenge for clinical implementation, primarily due to the expensive organoid culture media. However, Cure-GA, which utilizes a 384-pillar/well plate, requires only 30–40 μL of assay volume, thereby reducing the assay cost compared with conventional drug sensitivity tests that use 24- or 96-well plates requiring 100–600 μL of assay volume.^27^ To further reduce costs, the assay volume must be reduced to 10 μL in the 1536-well plate format. Second, we currently use surgical tissue for Cure-GA. To expand its applicability, Cure-GA could be compatible not only with surgical tissues but also with endoscopic tissues. However, since the smaller size of endoscopic tissues is a challenge for various drug screening, multiple samples should be required for endoscopic tissue sampling. Third, to validate the long-term performance of Cure-GA, a study comparing test results with RFS rates over 5 years is necessary in the future. Addressing these challenges is critical for enhancing the clinical utility and broader application of this system.

Seidlitz et al. suggested that GC organoids might serve as living biomarkers to predict therapeutic response and resistance in individual patients, thereby guiding personalized therapeutic approaches.^28^ In this study, we show that the Cure-GA platform could be a valuable tool for personalized cancer treatment strategies, especially for GC patients; however, it is necessary to collect and analyze long-term clinical data to establish broader clinical applications.

## Conclusion

In this study, we have developed a novel high-throughput chemotherapeutic drug screening system (Cure-GA) for GC patients. This system allows for rapid and automated evaluation of drug responsiveness using 3D culture-based tumoroids directly isolated from patients' tumor tissue. By leveraging this platform, clinicians can quickly identify effective chemotherapy treatments for individual GC patients, thus aiding in the formulation of optimal treatment strategies. Additionally, the development of an MPI-based prognosis prediction model further enhances the clinical utility of the Cure-GA platform by incorporating factors such as AUC, TNM stage, and tumor growth rate to predict patient outcomes. Overall, this study facilitates the translation of research findings into clinical practice, ultimately improving patient care and treatment outcomes in GC management.

## Supplementary Information

Supplementary file 1

Supplementary file 2

Supplementary file 3
