# Supplementary material for: High-Throughput Chemotherapeutic Drug Screening System for Gastric Cancer (Cure-GA)
Source: Ann Surg Oncol. 2025 Jan 23;32(5):3781–95. doi: 10.1245/s10434-024-16850-0 (PMC11976768; doi:10.1245/s10434-024-16850-0)
Supplement: Supplementary file 1 [file 10434_2024_16850_MOESM1_ESM.docx]

**Supplementary methods**

**Analysis of sequencing data.**

For whole-exome sequencing (WES) analysis, trimmed reads were mapped to the human reference genome GRCh38 using BWA-MEM2 (v2.2.1) ^1^ with the following parameters: -M, -T 0. Bam files were then sorted using SAMtools (v.1.16.1) ^2^. Duplication and base recalibration were conducted using GATK (v4.3.0.0; Picard v2.27.5) ^3^. For recalibration, Mills and 1000G gold standard indels, dbSNP (v138), and 1000G phase 1 high-confidence indels were used as known sites. To identify somatic mutations, single-nucleotide variants (SNVs) and small insertions and deletions (indels), we followed the GATK best practice guidelines. Briefly, variant calling was performed using MuTect2 with matched normal samples and gnomAD as a germline resource. The same matched normal samples were used for both the primary tumor and tumoroids. The raw variant calls were filtered using FilterMutectCalls. To minimize potential false-positive calls, the following filtering criteria were applied to qualified variants: 1) the number of read depths was at least 20 and 10 for tumor/tumoroid and normal samples, respectively, and 2) the alternative allele fraction was greater than 0.05. The variants that passed the filtering were annotated using Funcotator. The decomposition of variants was performed using Mutalisk, employing linear regression with mutation signatures identified in COSMIC stomach cancer ^4^. The CNA was estimated using CNVkit (v0.9.9) ^5^. Variant and CNA similarities between tumors and tumoroids were calculated by the Jaccard index and Pearson’s method from CNApp ^6^, respectively. The sequencing depth of each Bam file was calculated using mosdepth (v0.3.3) ^7^.

For mRNA analysis, Ribodetector (v0.2.7) with the ‘-e rrna’ option was used to exclude rRNA-derived reads that had not been depleted during library construction ^8^. After preprocessing, the reads were aligned against the human reference genome build GRCh38 with Homo_sapiens.GRCh38.108.gtf using STAR aligner (v2.7.10a) ^9^. The parameters for the aligner followed the Genomic Data Commons (GDC) pipeline (<https://docs.gdc.cancer.gov/Data/Bioinformatics_Pipelines/Expression_mRNA_Pipeline/>). Gene expression was quantified by RSEM (v1.3.1) ^10^; quantified counts were subsequently imported into DESeq2 (v.1.38.3) using tximport (v1.26.1) and normalized to variance-stabilizing transformation (VST) values ^11^. Overrepresentation analysis was performed with the hallmark gene set using WebGestalt ^12^.

NGSCheckMate (v1.0) was used for calculating the genotype correlation between WES/mRNA data.

**1.** Vasimuddin M, Misra S, Li H, Aluru S. Efficient Architecture-Aware Acceleration of BWA-MEM for Multicore Systems. Paper presented at: 2019 IEEE International Parallel and Distributed Processing Symposium (IPDPS); 20-24 May 2019, 2019.

**2.** Danecek P, Bonfield JK, Liddle J, et al. Twelve years of SAMtools and BCFtools. *GigaScience.* 2021;10(2):giab008.

**3.** Van der Auwera GA, O'Connor BD. *Genomics in the cloud: using Docker, GATK, and WDL in Terra.* O'Reilly Media; 2020.

**4.** Lee J, Lee Andy J, Lee J-K, et al. Mutalisk: a web-based somatic MUTation AnaLyIS toolKit for genomic, transcriptional and epigenomic signatures. *Nucleic Acids Research.* 2018;46(W1):W102-W108.

**5.** Talevich E, Shain AH, Botton T, Bastian BC. CNVkit: Genome-Wide Copy Number Detection and Visualization from Targeted DNA Sequencing. *PLOS Computational Biology.* 2016;12(4):e1004873.

**6.** Franch-Expósito S, Bassaganyas L, Vila-Casadesús M, et al. CNApp, a tool for the quantification of copy number alterations and integrative analysis revealing clinical implications. *eLife.* 2020/01/15 2020;9:e50267.

**7.** Pedersen BS, Quinlan AR. Mosdepth: quick coverage calculation for genomes and exomes. *Bioinformatics.* 2018;34(5):867-868.

**8.** Deng Z-L, Münch PC, Mreches R, McHardy AC. Rapid and accurate identification of ribosomal RNA sequences via deep learning. *Nucleic Acids Research.* 2022;50(10):e60-e60.

**9.** Dobin A, Davis CA, Schlesinger F, et al. STAR: ultrafast universal RNA-seq aligner. *Bioinformatics.* 2013;29(1):15-21.

**10.** Li B, Dewey CN. RSEM: accurate transcript quantification from RNA-Seq data with or without a reference genome. *BMC Bioinformatics.* 2011/08/04 2011;12(1):323.

**11.** Love MI, Huber W, Anders S. Moderated estimation of fold change and dispersion for RNA-seq data with DESeq2. *Genome Biology.* 2014/12/05 2014;15(12):550.

**12.** Liao Y, Wang J, Jaehnig EJ, Shi Z, Zhang B. WebGestalt 2019: gene set analysis toolkit with revamped UIs and APIs. *Nucleic Acids Research.* 2019;47(W1):W199-W205.
