# Supplementary material for: High-Throughput Chemotherapeutic Drug Screening System for Gastric Cancer (Cure-GA)
Source: Ann Surg Oncol. 2025 Jan 23;32(5):3781–95. doi: 10.1245/s10434-024-16850-0 (PMC11976768; doi:10.1245/s10434-024-16850-0)
Supplement: Supplementary file 3 [file 10434_2024_16850_MOESM3_ESM.pptx]

## Slide 1
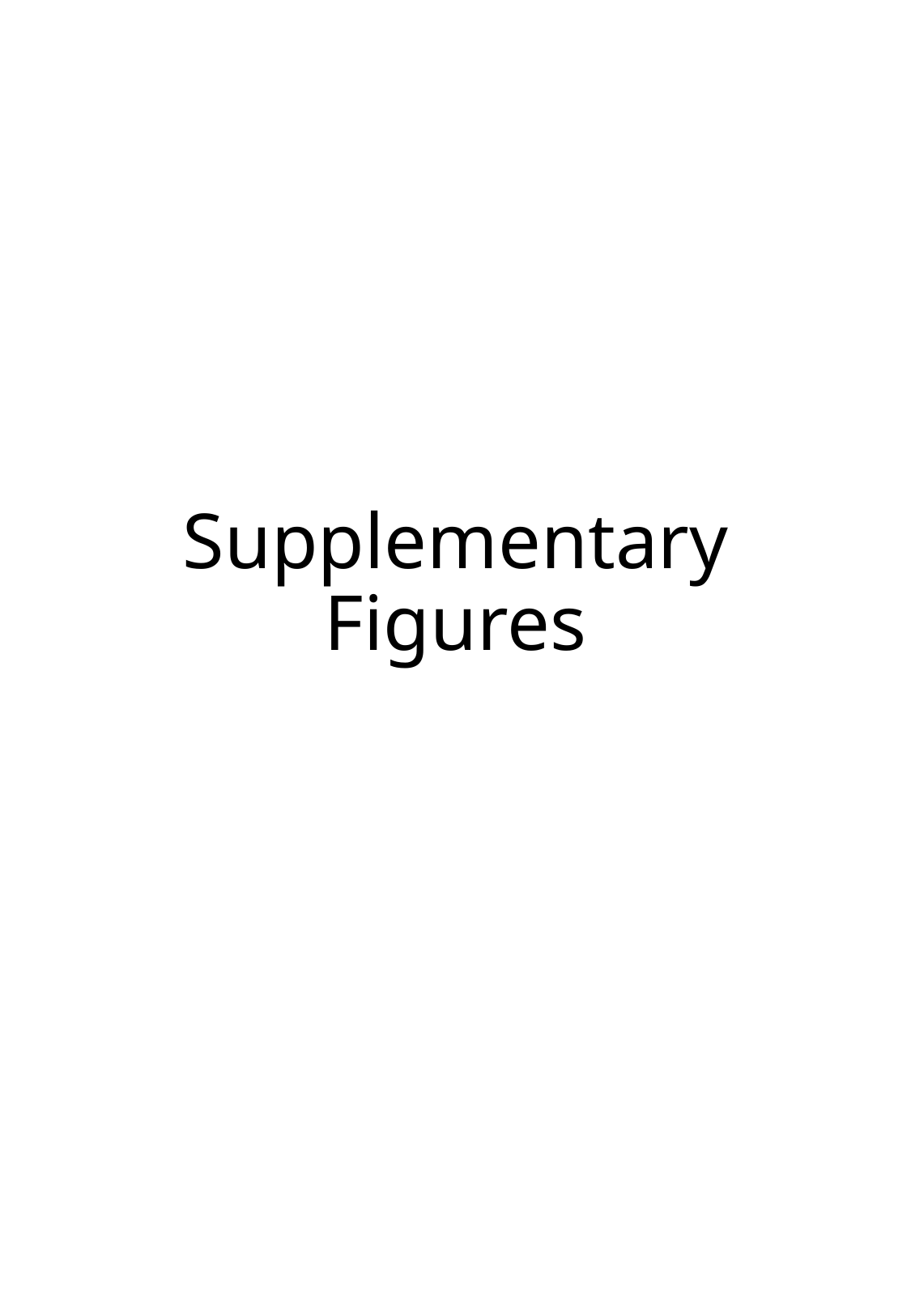

# Supplementary Figures

## Slide 2
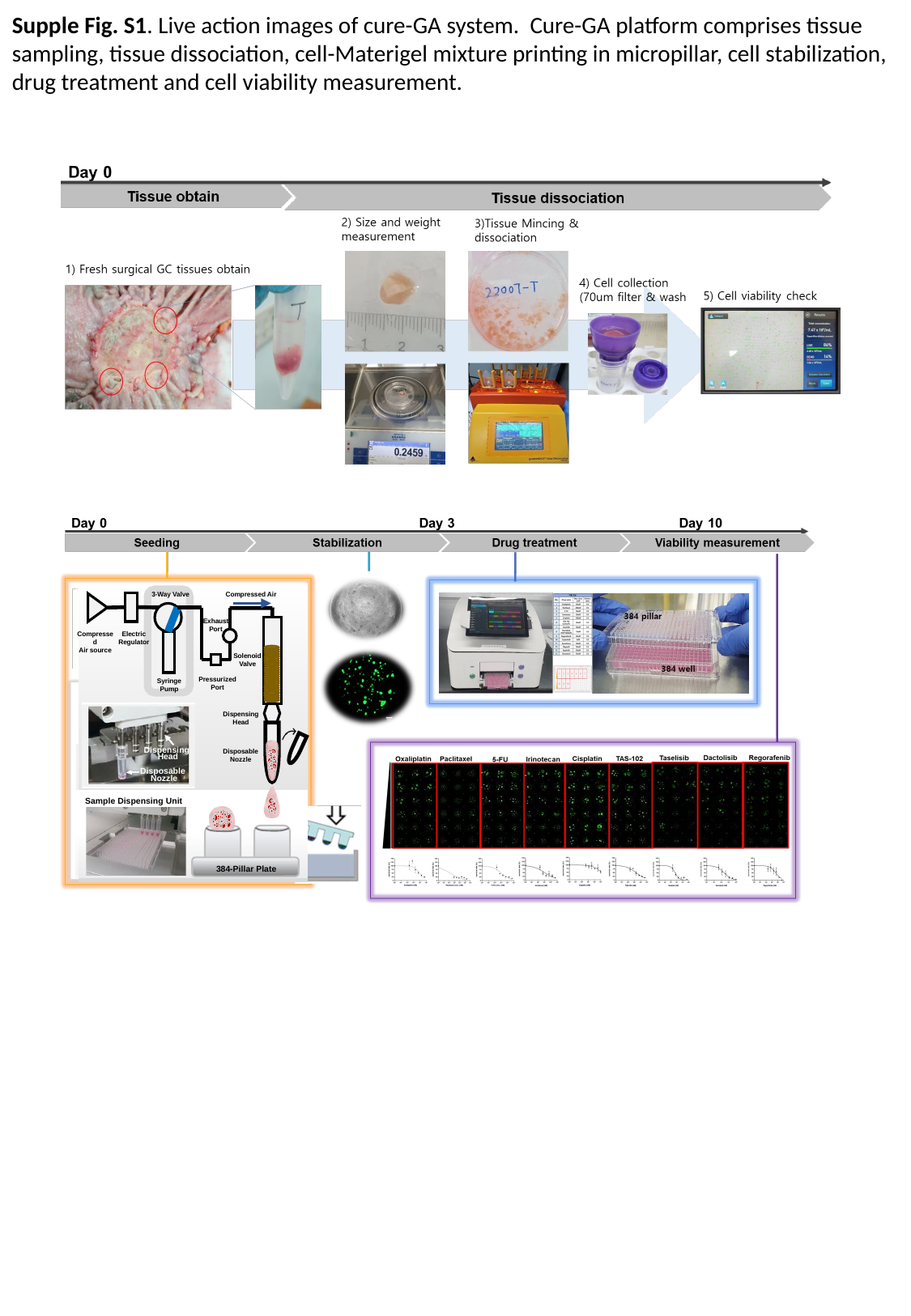

Supple Fig. S1. Live action images of cure-GA system. Cure-GA platform comprises tissue sampling, tissue dissociation, cell-Materigel mixture printing in micropillar, cell stabilization, drug treatment and cell viability measurement.
Compressed Air
3-Way Valve
Exhaust
Port
Compressed
Air source
Electric
Regulator
Solenoid
Valve
Pressurized
Port
Syringe
Pump
Dispensing
Head
Disposable
Nozzle
Dispensing
Head
Disposable
Nozzle
Sample Dispensing Unit
384-Pillar Plate

## Slide 3
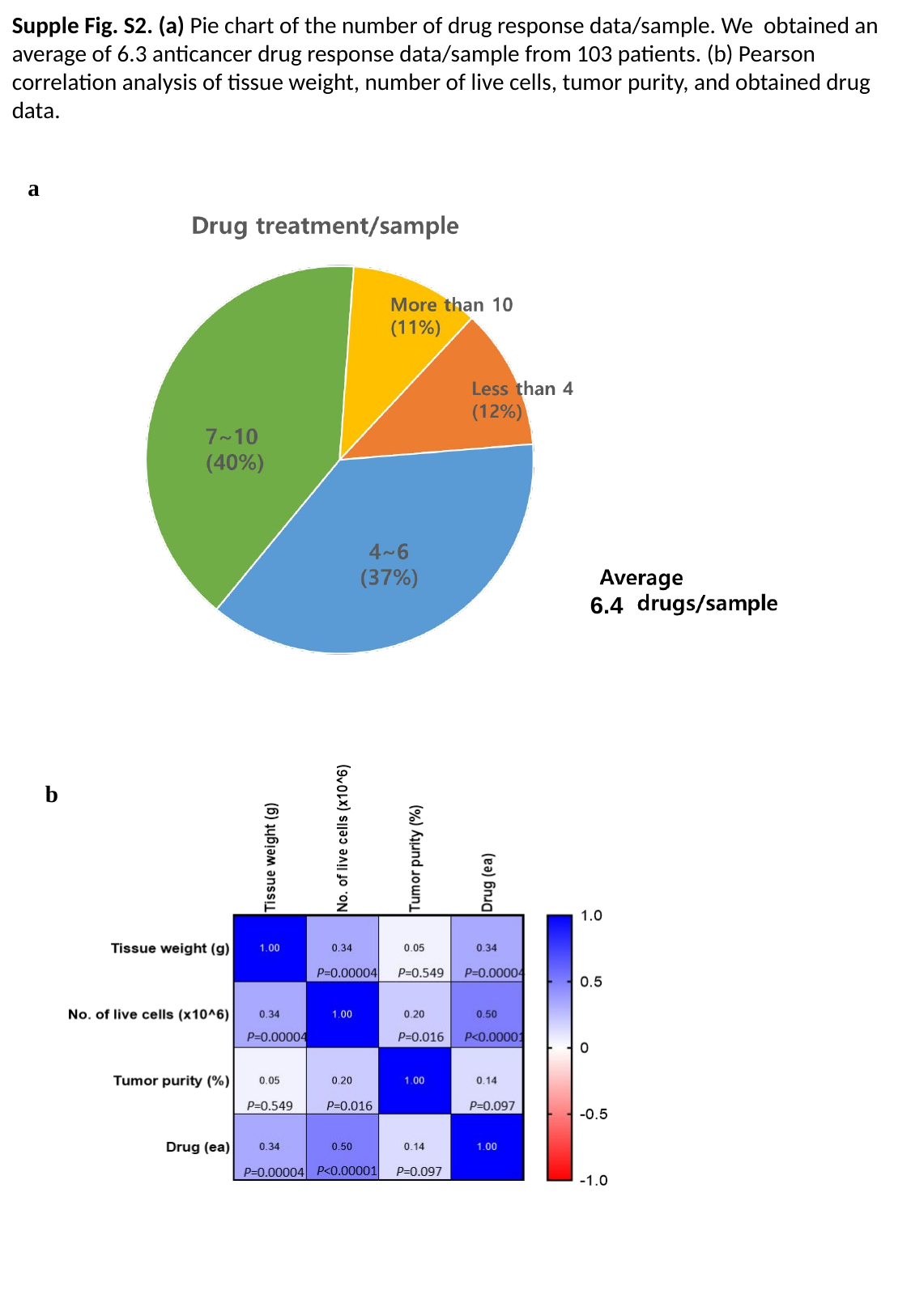

Supple Fig. S2. (a) Pie chart of the number of drug response data/sample. We obtained an average of 6.3 anticancer drug response data/sample from 103 patients. (b) Pearson correlation analysis of tissue weight, number of live cells, tumor purity, and obtained drug data.
a
6.4
b

## Slide 4
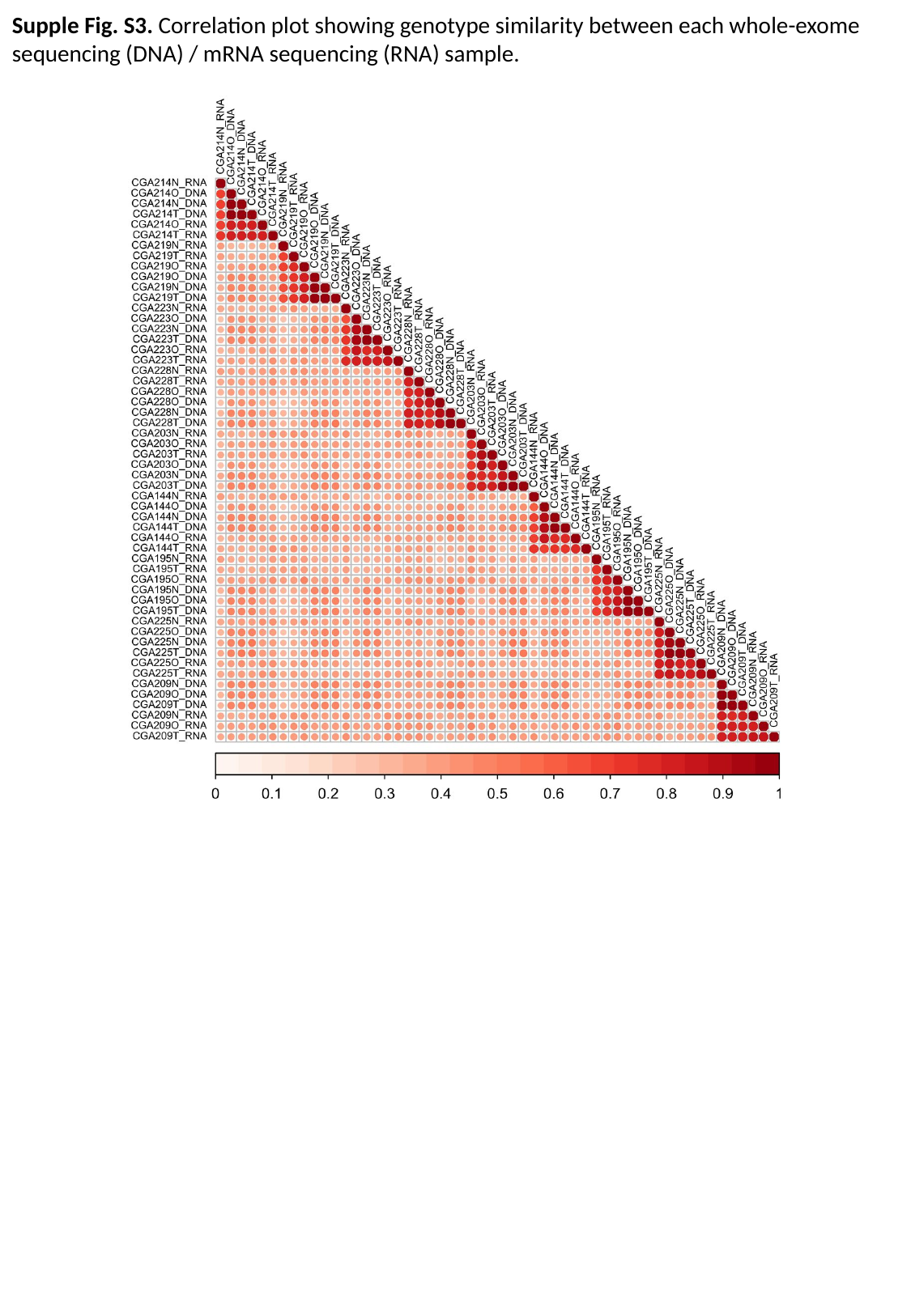

Supple Fig. S3. Correlation plot showing genotype similarity between each whole-exome sequencing (DNA) / mRNA sequencing (RNA) sample.

## Slide 5
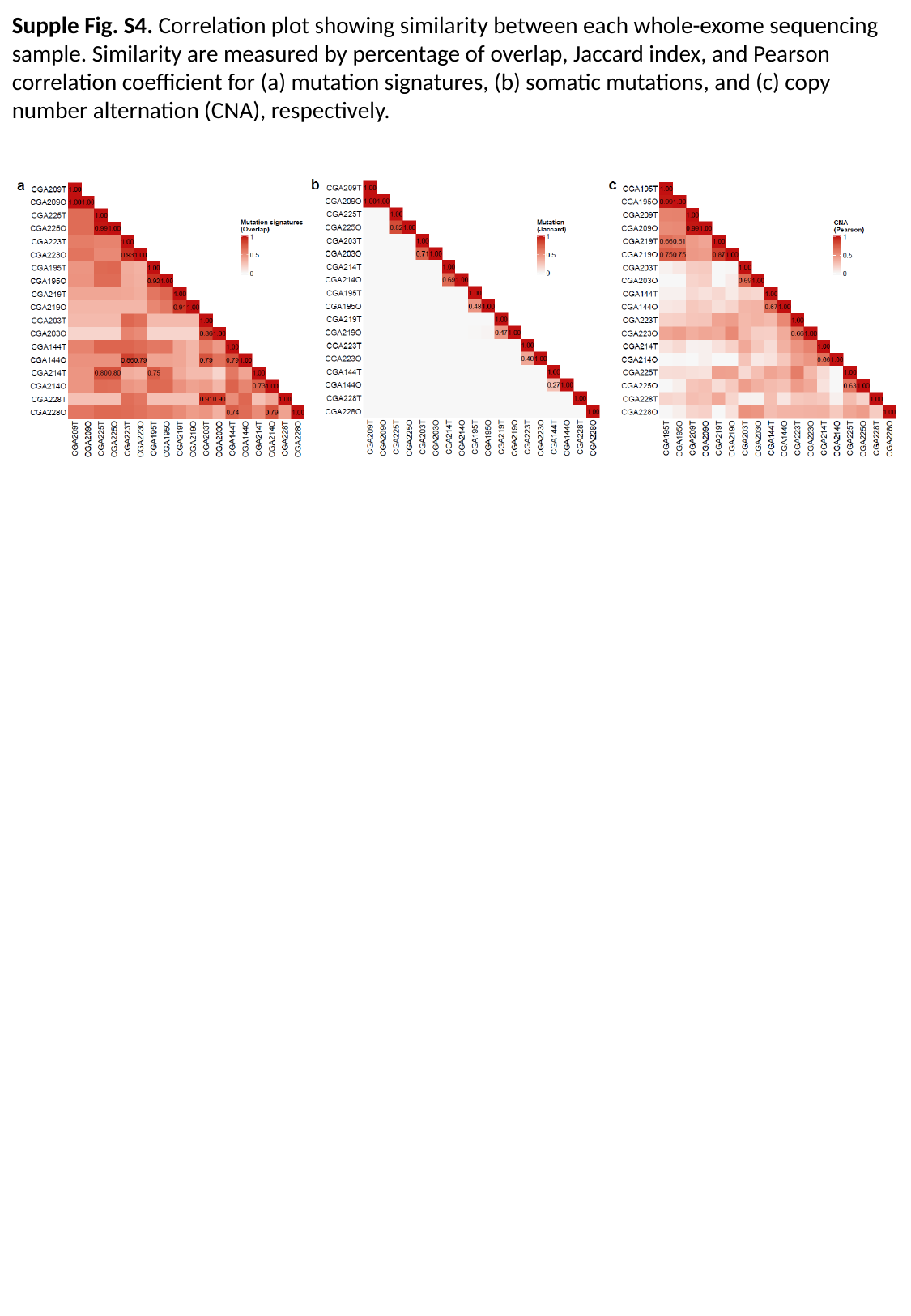

Supple Fig. S4. Correlation plot showing similarity between each whole-exome sequencing sample. Similarity are measured by percentage of overlap, Jaccard index, and Pearson correlation coefficient for (a) mutation signatures, (b) somatic mutations, and (c) copy number alternation (CNA), respectively.

## Slide 6
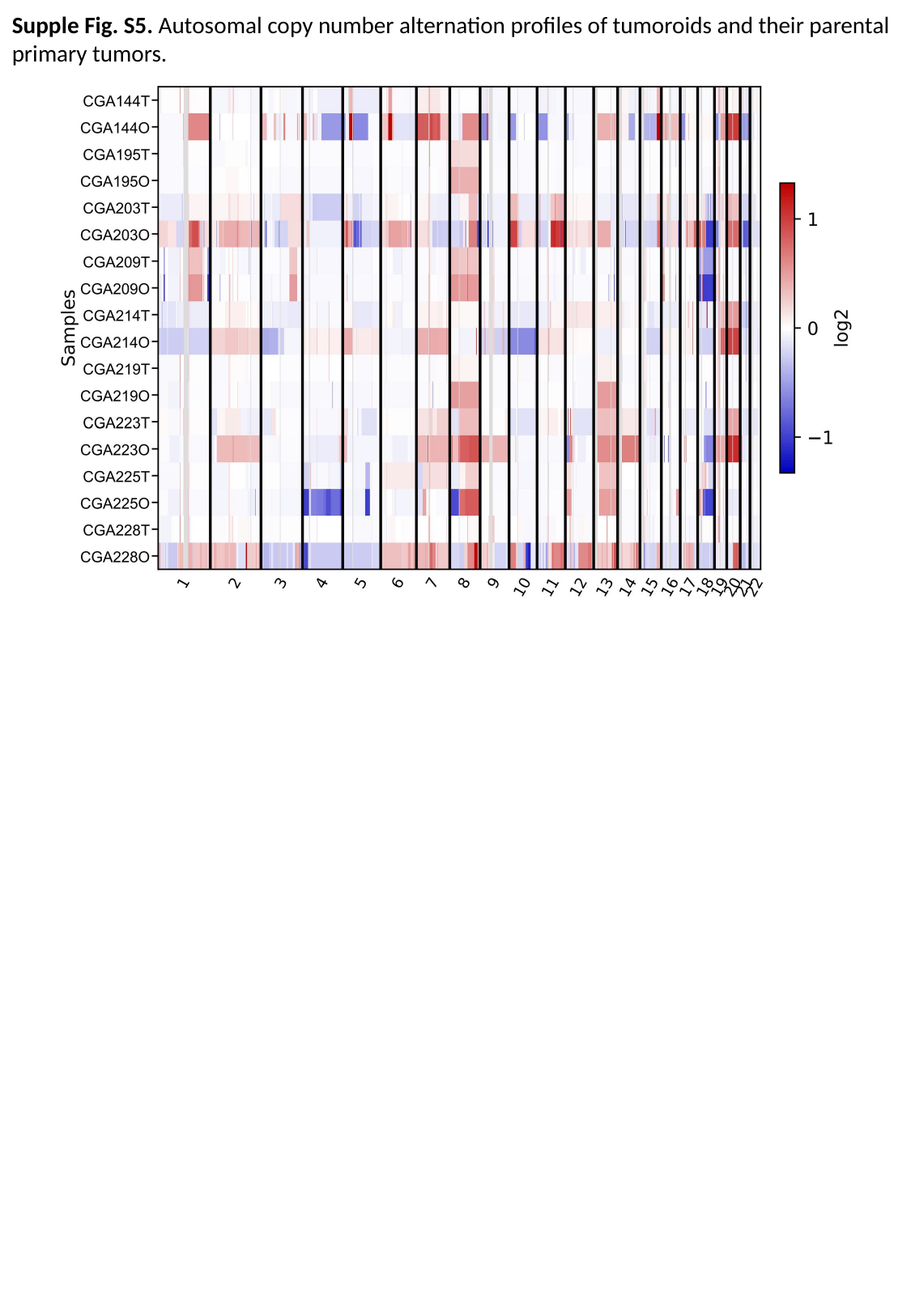

Supple Fig. S5. Autosomal copy number alternation profiles of tumoroids and their parental primary tumors.

## Slide 7
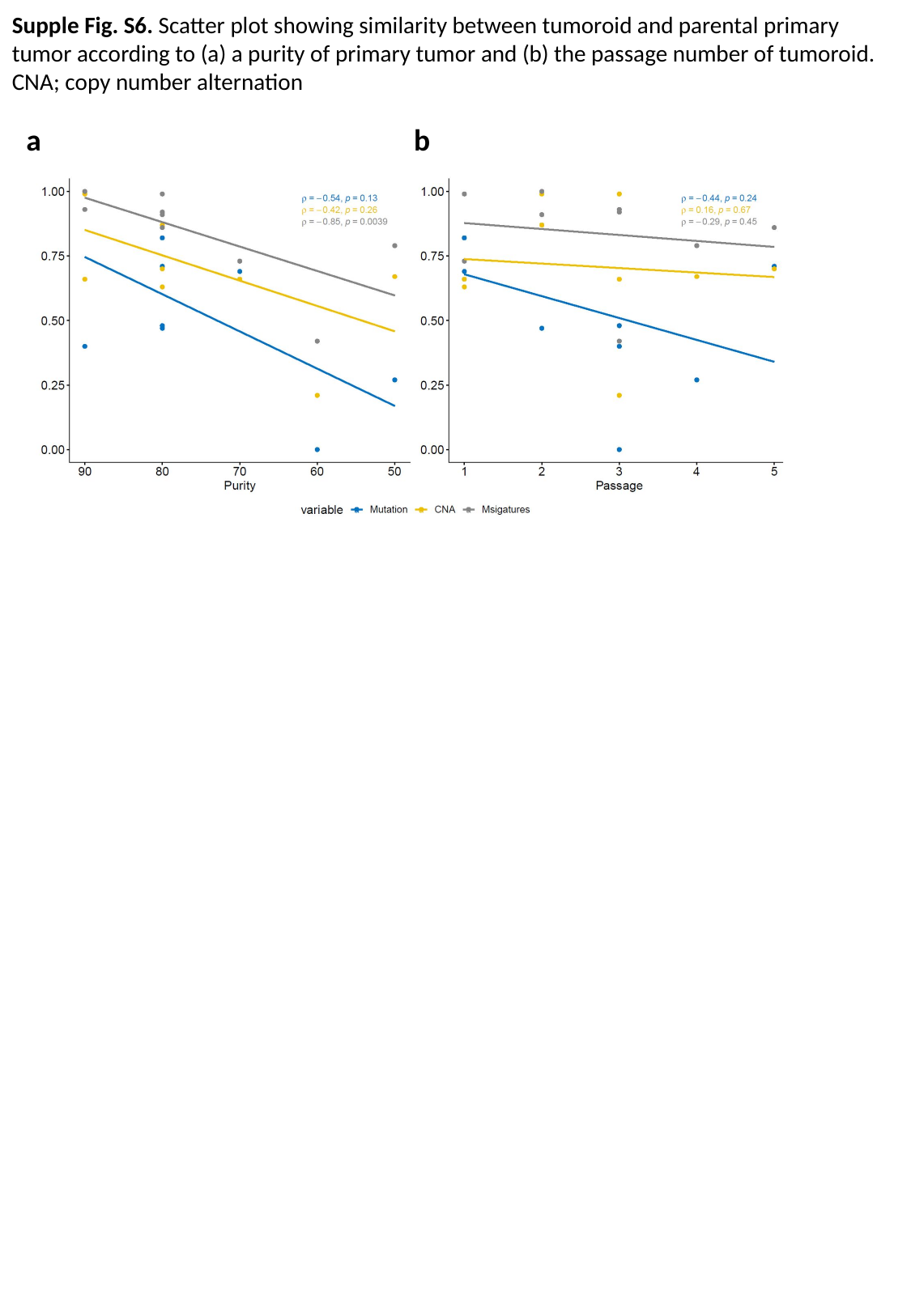

Supple Fig. S6. Scatter plot showing similarity between tumoroid and parental primary tumor according to (a) a purity of primary tumor and (b) the passage number of tumoroid. CNA; copy number alternation
a
b

## Slide 8
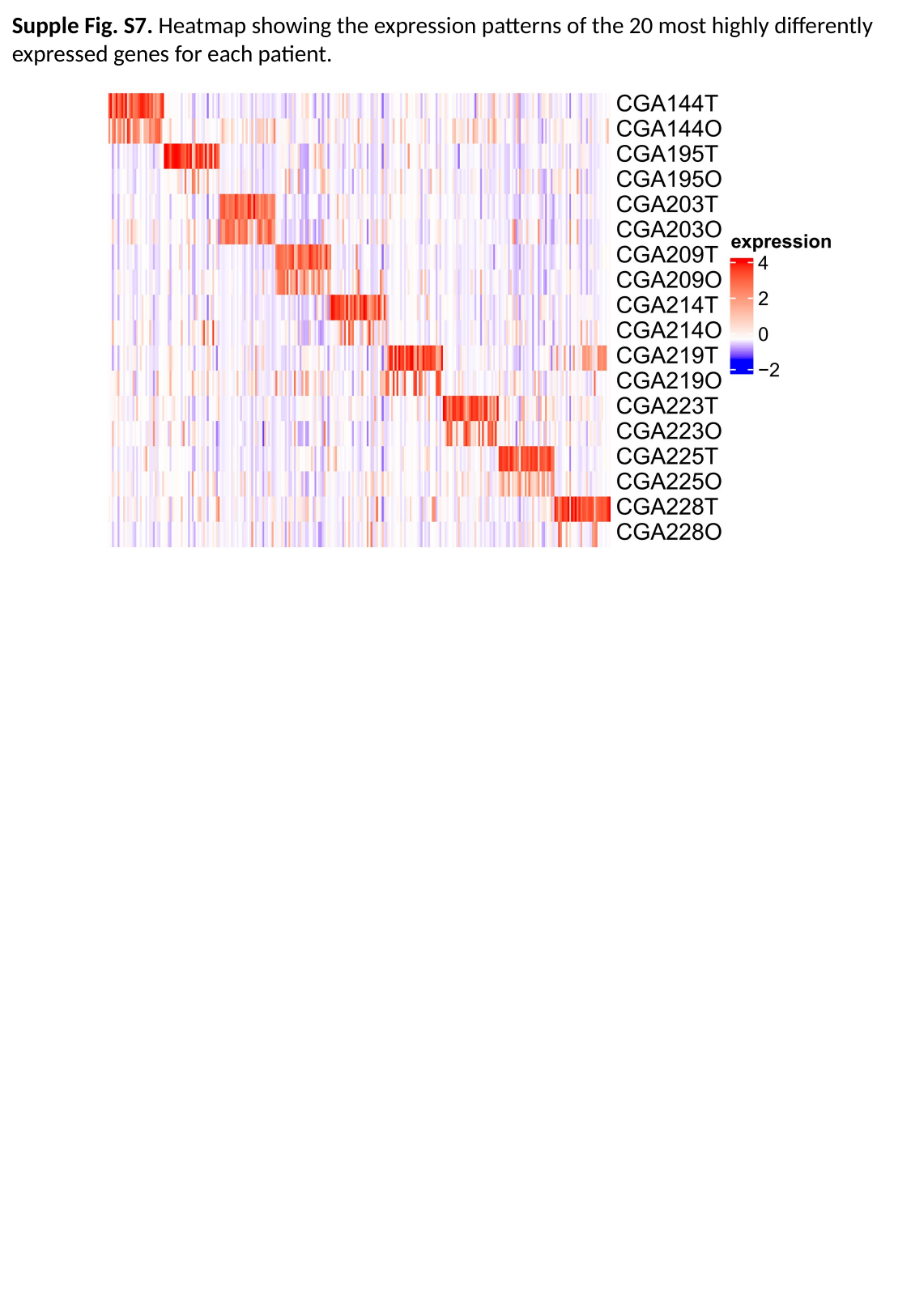

Supple Fig. S7. Heatmap showing the expression patterns of the 20 most highly differently expressed genes for each patient.

## Slide 9
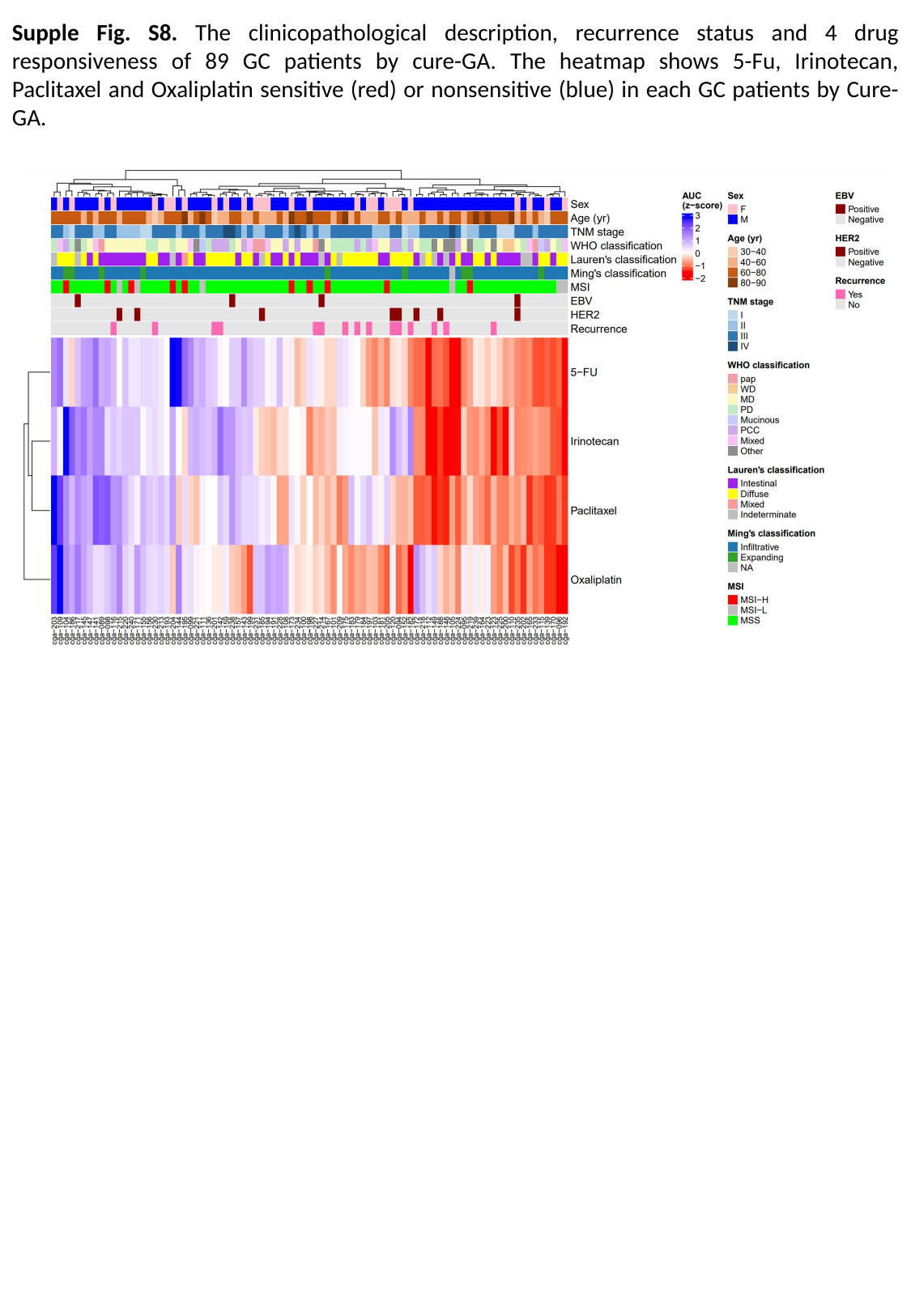

Supple Fig. S8. The clinicopathological description, recurrence status and 4 drug responsiveness of 89 GC patients by cure-GA. The heatmap shows 5-Fu, Irinotecan, Paclitaxel and Oxaliplatin sensitive (red) or nonsensitive (blue) in each GC patients by Cure-GA.

## Slide 10
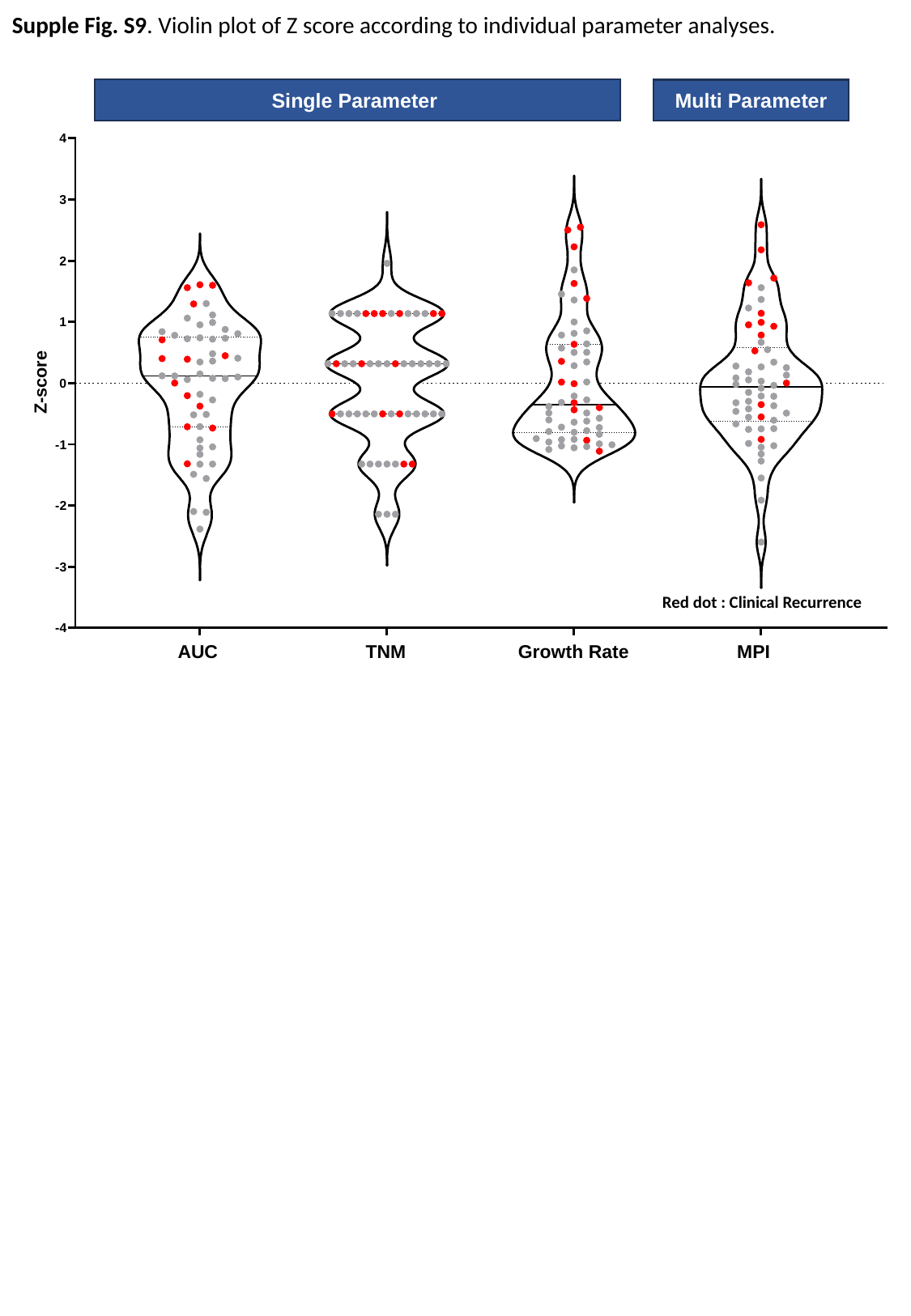

Supple Fig. S9. Violin plot of Z score according to individual parameter analyses.
Single Parameter
Multi Parameter
TNM
Growth Rate
AUC
Red dot : Clinical Recurrence
MPI
